# Tuning Nb Solubility, Electrical Properties, and Imprint through PbO Stoichiometry in PZT Films

**DOI:** 10.3390/ma16113970

**Published:** 2023-05-25

**Authors:** Betul Akkopru-Akgun, Susan Trolier-McKinstry

**Affiliations:** 1 Center for Dielectrics and Piezoelectrics, Materials Research Institute, The Pennsylvania State University, University Park, PA 16802, USA; set1@psu.edu; 2 Department of Materials Science and Engineering, The Pennsylvania State University, University Park, PA 16802, USA

**Keywords:** heavily Nb doped PZT films, charge compensation, imprint

## Abstract

Lead zirconate titanate (PZT) films with high Nb concentrations (6–13 mol%) were grown by chemical solution deposition. In concentrations up to 8 mol% Nb, the films self-compensate the stoichiometry; single phase films were grown from precursor solutions with 10 mol% PbO excess. Higher Nb concentrations induced multi-phase films unless the amount of excess PbO in the precursor solution was reduced. Phase pure perovskite films were grown with 13 mol% excess Nb with the addition of 6 mol% PbO. Charge compensation was achieved by creating lead vacancies when decreasing excess PbO level; using Kroger-Vink notation, $Nb_{Ti}^{•}$ are ionically compensated by $V_{Pb}^{\prime \prime }$ to maintain charge neutrality in heavily Nb-doped PZT films. With Nb doping, films showed suppressed {100} orientation, the Curie temperature decreased, and the maximum in the relative permittivity at the phase transition broadened. The dielectric and piezoelectric properties were dramatically degraded due to increased quantity of the non-polar pyrochlore phase in multi-phase films; $\varepsilon_{r}$ reduced from 1360 ± 8 to 940 ± 6, and the remanent d_33,f_ value decreased from 112 to 42 pm/V when increasing the Nb concentration from 6 to 13 mol%. Property deterioration was corrected by decreasing the PbO level to 6 mol%; phase pure perovskite films were attained. ε_r_ and the remanent d_33,f_ increased to 1330 ± 9 and 106 ± 4 pm/V, respectively. There was no discernable difference in the level of self-imprint in phase pure PZT films with Nb doping. However, the magnitude of the internal field after thermal poling at 150 °C increased significantly; the level of imprint was 30 kV/cm and 11.5 kV/cm in phase pure 6 mol% and 13 mol% Nb-doped films, respectively. The absence of mobile $V_{O}^{••},$ coupled with the immobile $V_{Pb}^{\prime \prime }$ in 13 mol% Nb-doped PZT films, leads to lower internal field formation upon thermal poling. For 6 mol% Nb-doped PZT films, the internal field formation was primarily governed by (1) the alignment of ${\left(V_{Pb}^{\prime \prime }-V_{O}^{••}\;\right)}^{x}$ and (2) the injection and subsequent electron trapping by Ti^4+^. For 13 mol% Nb-doped PZT films, hole migration between $V_{Pb}^{\prime \prime }$ controlled internal field formation upon thermal poling.

## 1. Introduction

Lead zirconate titanate (PZT) films have been widely used in many piezoelectric microelectromechanical systems (piezoMEMS), including actuators, transducers, sensors, ink-jet printers, and energy harvesters [1,2,3,4]. These applications demand films with high piezoelectric performance that withstand significant electrical and temperature stresses during their use. The piezoelectric properties of PZT films strongly depend on their Zr/Ti ratio, annealing atmosphere, crystal orientation, and doping profile. PZT compositions near the morphotropic phase boundary with a Zr/Ti ratio of 52/48 have high piezoelectric coefficients and relative permittivities due to the coexistence of tetragonal and rhombohedral phases [5,6]. The degree of preferred orientation in crystallites also affects polarization; the remanent polarization, P_r_, was shown to increase linearly with increased fractions of c-oriented grains in {100}-oriented films [7,8].

The piezoelectric properties and reliability can be further controlled by solid solutions, i.e., acceptors (Mn^2+/3+/4+^, Fe^2+/3+/4+^, or Mg^2+^ on the B-site), [9,10,11] isovalent ions (Sr^2+^ or Ba^2+^ on the A site), [12,13] and donors (La^3+^ substituting for Pb^2+^ on the A site, or Nb^5+^, Ta^5+^, or Sb^5+^ on the B-site) [14,15,16]. Isovalent doping decreases the Curie temperature, T_C_, and, therefore, increases the room temperature dielectric permittivity. The substitution of acceptor ions into PZT introduces defect dipoles associated with the charge compensating oxygen vacancies that suppress domain wall motion [17,18], decreasing piezoelectric and dielectric responses and increasing the mechanical quality factor. Acceptor ions, together with oxygen vacancies, form defect dipoles ${\left(A_{Ti}^{\prime \prime }-V_{O}^{••}\;\right)}^{\prime \prime }$, which contribute to the formation of internal fields [19,20]. These fields enhance the stability of the dielectric and piezoelectric properties and reduce the aging rate [20]. Addition of donor ions into PZT films, on the other hand, diminishes the concentration of oxygen vacancies, $\left[V_{O}^{••}\right]$, due to PbO evaporation during heat treatment. This increases domain wall mobility, enhancing permittivity and the piezoelectric coefficient. Decreased oxygen vacancy concentration also reduces the leakage current rise and improves the film lifetime upon electrical degradation under DC electric fields [21]. Charge compensation in donor doped PZT films is achieved by the creation of $V_{Pb}^{\prime \prime }$ or electrons. Haccart et al. reported that Nb content in PZT films increases self-polarization due to the alignment of ${\left(V_{Pb}^{\prime \prime }-Nb_{Ti}^{•}\;\right)}^{\prime }$ [22]. If such defect dipoles do exist, they presumably orient during the film processing, as they are unlikely to be mobile at room temperature under realizable electric fields.

The influence of Nb doping on the dielectric and piezoelectric properties of PZT films has been extensively studied; however, the literature reports contradictions and discrepancies in optimal Nb concentration. Kim et al. showed that the relative dielectric constant, $\varepsilon_{r},$ increased from 1100 to 1580 when undoped PZT films were replaced by 2 mol% Nb doped PZT films [23]. Increasing the Nb levels to 4 mol% decreased $\varepsilon_{r}$ to 1300 due to the formation of a non-ferroelectric phase. There are multiple studies reporting that $\varepsilon_{r}$ and piezoelectric properties were maximized in 2 mol% Nb-doped PZT films [24,25]. Souza et al. observed improvement in dielectric and piezoelectric properties of PZT films with 1% Nb [26]. However, Pavio-Santos et al. found that T_c_ decreased with increasing Nb concentration to 10 mol%; dielectric properties began to degrade at Nb levels > 5 mol% [27]. Kurchania and Milne observed a minimum coercive field, E_c_, and maximum ferroelectric properties in 3 to 4 mol% Nb-doped PZT films [28]. Araujo et al. obtained the highest remanent polarization with the addition of 5 mol% Nb into PZT films [29]. Nb solubility limits influenced the percentage of Nb needed for superior dielectric and piezoelectric properties as the formation of a non-ferroelectric pyrochlore phase weakened film properties.

The extent of Nb solubility in PZT ceramics is reported to be 6–7 mol% [30,31,32,33]. Pereira et al. observed perovskite phase formation up to 7 mol% Nb in PZT (65/35) ceramics, beyond which a secondary pyrochlore phase was formed [30]. Similar solubility limits were reported by Chu et al. and Mohiddon et al. for PZT (52/48) and PZT (65/35) ceramics [31,32]. The solubility of Nb in sol-gel-derived PZT films, however, varied between 2–10 mol% [26,28,33,34]. Li et al. demonstrated perovskite phase formation up to 4% Nb in PZT (52/48) films [33] Likewise, Kim et al. found that the addition of Nb concentrations > 5 mol% led to pyrochlore formation in PZT films [23]. Lower solubility limits of Nb in PZT (40/60) films were also reported; in one publication, a phase mixture of perovskite and pyrochlore was observed at 1 mol% Nb [26]. The minimum temperature required to obtain single phase perovskite films increased from 600 to 700 °C by increasing Nb levels from 2 to 5 mol% [28]. Pyrochlore free PZT films were reported for up to 10 mol% Nb with annealing at 650 °C [34].

Pyrochlore formation and its stability are controlled by the valence state and ionic radii of the B-site cations, as well as Nb and Pb content [34,35,36]. There are two distinct types of pyrochlore (or fluorite, if the oxygen vacancies are disordered) in PZT: (1) a transient pyrochlore, represented by the chemical formula A_2_B_2_O_7_ (A_2_B_2_O_6_O′) and (2) a lead deficient pyrochlore, Pb_2−x_(Zr,Ti)_2_O_6−x_. In the fluorite structure, three different oxygen sites are present, 48f with two A and B near neighbors, 8b positions with 4 A near neighbors, and 8a positions with 4 B near neighbors (Figure S1). The 8a positions are unoccupied in the structure of pyrochlore, thus, near neighbor 4 B cations are electrostatically shielded from each other by the displacement of x of 48f oxygens towards the neighboring B cations [37,38]. The 48f oxygen initially located at x = 0.375 shift to x = 0.3125, where B cations are in corner shared octahedra along the [110]. A lead-deficient pyrochlore phase can form as a result of PbO evaporation during annealing. The formation and retention of the pyrochlore phase is often influenced by the valence state and ionic radii of the B-site cations. Subramanian et al. [37] found that the 48f oxygen x parameter increased with increasing cation size; when it exceeded 0.33, the pyrochlore structure dominated. Klissurska et al. observed enhanced pyrochlore formation in Zr-rich PZT regions, since the x parameter was 0.375 for Zr^4+^ [34].

When Nb is substituted into the PZT lattice, an A-site vacancy is required to maintain electroneutrality by ionic compensation, as described using Kroger-Vink notation in Equation (1). The solubility of Nb ions can be improved by creating lead vacancies in the

(1) $$2\left[V_{O}^{••}\right]+[Nb_{Ti}^{•}]+\left[h^{•}\right]=2\left[V_{Pb}^{\prime \prime }\;\right]+\left[e^{\prime }\right]$$

perovskite lattice. M’peko et al. found that the solubility limit of Nb in PZT 65/35 ceramics, which is around 7 mol%, decreased to 4 mol% when the excess PbO content increased from 0 to 4 mol% [39]. In the pyrochlore structure, on the other hand, a change in oxygen stoichiometry, or formation of an A-site vacancy, enables electroneutrality. Thus, variations in either the Nb concentration or the Pb content affect the stability of the pyrochlore phase [35,38,40].

Unlike sol-gel-derived PZT films or/and bulk ceramics, much higher solubility limits for Nb have been reported in sputtered PZT films. Fujii et al. acquired pyrochlore free at 13 mol% Nb-doped PZT films with e_31,f_ exceeding −25 µC/cm^2^, considerably above the values most often reported for well oriented PZT films (~−20 C/m^2^) [41]. Similarly, Berenov et al. observed enhancement of the pyroelectric coefficient in PZT (30/70) films with 12 mol% Nb doping, together with an increased photovoltaic effect [42].

In addition, many heavily Nb-doped PZT films also exhibit strong imprint; they are self-polarized without an additional poling. For example, it has been reported that polarization loss in sputtered PZT films with 13 mol% Nb at 150–260 °C is drastically decreased due to the presence of a strong internal field that reduces back-switching of ferroelectric domains [43]. The high internal field in the pristine state, coupled with the low oxygen vacancy concentration, allow these films to operate at high temperatures without degradation in piezoelectric and pyroelectric properties. Two different mechanisms explain the formation of built-in potential in pristine films: (1) alignment of ${\left(V_{Pb}^{\prime \prime }-Nb_{Ti}^{•}\;\right)}^{\prime }$ defect dipoles by a residual stress gradient along the film thickness [44] and (2) alignment of oppositely charged defects, such as $V_{Ti,Zr}^{\prime \prime \prime \prime },\;\mathrm{and}\;Nb_{Ti}^{•}$ [42].

As the contradictory results in the literature suggest, the charge compensation mechanism of Nb and physical origin of self-imprint in these films are not yet fully understood. The synthesis of heavily Nb doped PZT films via sputtering has been reported; the processing of pyrochlore-free heavily (13 mol%) Nb-doped PZT films via chemical solution deposition has been reported sparsely, and it remains a challenge. Recently, pyrochlore-free Nb-doped PZT films up to 13 mol% were produced via chemical solution deposition, and the charge transport mechanisms that control DC electrical degradation and lifetime were reported [45]. However, as detailed above, numerous questions remain regarding these highly doped films. Of particular interest is whether or not all heavily Nb-doped films will behave the same way. The extent of substitutional solid solution needs to be assessed by varying the lead content. In addition, the mechanisms that control self-imprint in these films need to be studied in detail.

Thus, in this study, pyrochlore-free, 13 mol% Nb-doped PZT films were synthesized via chemical solution deposition. The excess PbO level in the PNZT solution was systematically decreased to introduce lead vacancies that charge-compensate the Nb ions. The microstructural and electrical effects of elevated (>6 mol%) of Nb were investigated as a function of the amount of excess PbO in the deposited chemical solution in Pb_1+y_(Zr_0.52_Ti_0.48_)_1−x_Nb_x_O_3_, x = 0.06–0.13, y = 0–0.1. The effect of y on the solubility limit of Nb and the stability of the pyrochlore phase in the perovskite lattice was explored by X-ray diffraction and Field Emission Scanning Microscopy (FESEM). Dielectric, ferroelectric, and piezoelectric properties were investigated in pristine state and after poling under an electric field of 250 kV/cm for 30 min at 180 °C using polarization—electric field (P-E) hysteresis measurements, LCR meter, and a Double Beam Laser Interferometer (DBLI). Variations in the dielectric and ferroelectric properties of the film were related to the structure and microstructure. Additionally, charge compensation and the self-imprint mechanism were studied via Charge-Based Deep Level Transient Spectroscopy (Q-DLTS), as well as Thermally Stimulated Depolarization Current (TSDC) measurements.

## 2. Experimental Procedure

### 2.1. Solution Preparation

Heavily Nb-doped PZT thin film solutions with Zr/Ti composition of 52/48 were prepared via the sol-gel method using lead acetate trihydrate (Pb(CH_3_CO_2_)_2_ • 3H_2_O, Sigma-Aldrich), zirconium (IV) propoxide (Zr(OCH_2_CH_2_CH_3_)_4_, Sigma-Aldrich), titanium (IV) isopropoxide (Ti(OCH(CH_3_)_2_)_4_, Sigma-Aldrich), niobium (V) ethoxide (Nb(OCH_2_CH_3_)_5_, Sigma-Aldrich), 2-methoxyethanol (CH_3_OCH_2_CH_2_OH, Sigma-Aldrich), acetylacetone (CH_3_COCH_2_COCH_3_, Sigma-Aldrich), and acetic acid (CH_3_CO_2_H, Sigma-Aldrich). Two solution series were prepared: (1) 6–17 mol% Nb-doped PZT with 10 mol% excess PbO and (2) 13 mol% Nb-doped PZT with varying excess PbO level from 10 to 0 mol%. The excess PbO level was decreased systematically in the solution to enhance Nb solubility in PZT through the formation of lead vacancies. The Nb concentration in the solution was estimated as Pb_1+y_$\left({\mathrm{Zr}}_{0.52\left(1-x\right)}{\mathrm{Ti}}_{0.48\left(1-x\right)}{\mathrm{Nb}}_{x}\right)O_{3}$, x = 0.06–0.13; the amount of excess PbO in the solution, y, was changed from 0–0.1.

A detailed description of the solution preparation was previously reported [20]. Briefly, lead acetate trihydrate was mixed with 120 mL of 2-methoxyethanol (2-MOE) in a glass beaker under Ar at 120 °C. Then, the solution mixture was dehydrated under vacuum until it precipitated completely. In another flask, titanium iso-propoxide and zirconium n-propoxide were mixed and stirred in 150 mL of 2-MOE for 30 min. This solution mixture was gradually added to the lead powder and refluxed for 2 h under Ar at 120 °C. The final solution mixture was distilled under vacuum and diluted with 22.5 vol% of acetylacetone and 5 vol% of 2-MOE to obtain 0.4 M. The PZT solution is expected to have a viscosity of 2–5 cP [46,47].

### 2.2. Coating Process

The solution was first dispensed on to silicon substrates with a SiO_2_ barrier layer, 200 Å of Ti, and 1500 Å of Pt (Nova Electronic Materials, Inc., Richardson, TX, USA) using a 0.1 μm polytetrafluoroethylene (PTFE, Restek, Bellefonte, PA, USA) syringe filter, and then it was spun at 150 rpm for 30 s. After each layer deposition, the film was pyrolyzed at 250 °C and 450 °C for 3 min, respectively. Then, the PZT film layer was annealed at 700 °C for 60 s by rapid thermal annealing (RTA) under 10 SLPM O_2_ flow. This entire process was reiterated until a thickness of 400 nm was achieved. Lastly, a PbO layer was overcoated onto the PZT film to compensate for PbO loss upon annealing. A 4M acetic acid solution was used to remove residual PbO. The thermal decomposition of a PZT gel with a similar Zr/Ti ratio was reported in the literature. While physically adsorbed water was removed at temperatures lower than 200 °C, the vast majority of organic and volatiles were removed at 200–500 °C [46].

### 2.3. Materials Characterization

The crystal structure and orientation of the PZT films were determined using a X’Pert Pro MPD diffractometer (PANalytical, Almelo, The Netherlands) using a Cu *Kα* X-ray source at 40 kV. X-ray diffraction patterns between 20° and 60° 2θ were attained using a step size of 0.02° and holding time of 60 s.

The surface and cross section morphology of the films was investigated via a G500 model FESEM (ZEISS Gemini SEM, Jena, Germany) in secondary electron imaging mode at 5 kV.

To perform electrical characterization, 200 μm to 1 mm diameter circular Pt top electrode arrays were patterned on the PZT films using double-layer photolithography and lift-off processing. Sizes of ~100 nm-thick Pt films were sputtered on the PZT film (CMS-Sputter System, Kurt J. Lesker Company, Pittsburgh, PA, USA), on top of the patterned photoresist. The Pt top electrodes were post-heat treated at 650 °C for 1 min in a RTA after liftoff. The film was etched using a buffered oxide etchant (10:1 BOE, a mixture of ammonium fluoride (NH_4_F) and hydrofluoric acid (HF)) at room temperature to reach the bottom Pt electrode. The dielectric properties of the films were explored at 1 kHz with an oscillation voltage of 0.03 V, using a LCR meter (Hewlett-Packard 4284A Precision, Agilent Technologies, Inc., Palo Alto, CA, USA). Polarization—electric field, P-E, measurements were performed via a Precision Multiferroic Analyzer (Radiant Technologies, Inc., Albuquerque, NM, USA) with a triangular waveform at 100 Hz. The Rayleigh measurements of the permittivity were conducted at 10^2^–10^5^ Hz using an AC electric field of 0.5–25 kV/cm to explore dielectric non-linearities at sub-switching fields. In the Rayleigh regime, where the permittivity linearly increases with increasing AC electric field, the $\varepsilon_{r}$ can be described as follows:

(2) $$\varepsilon_{r}=\varepsilon_{init}+\alpha \cdot E_{AC}$$

where $\varepsilon_{init}$ represents dielectric response due to intrinsic lattice contribution and reversible domain wall motion, and α defines the contribution of irreversible domain wall motion.

The effective longitudinal piezoelectric coefficient (d_33,f_) was measured via double beam laser interferometry (AixACCT DBLI). The PZT films were poled at 180 °C under a DC electric field of 250 kV/cm for 30 min using a HP 6200B DC power supply (Agilent Technology, Palo Alto, CA, USA) before taking DBLI measurements. The remanent d_33,f_ was determined via extrapolating d_33,f_ signal back to E_DC_ = 0 V. The longitudinal piezoelectric coefficient is the proportionality constant that relates longitudinal deformation to the electric field applied along the poling direction.

To assess variation in defects and trap sites in heavily Nb doped PZT films, Q-DLTS measurements were conducted between 300–800 K. The amplitude of the Q-DLTS signals can be expressed as a charge difference extracted between two distinct time periods (discharging duration), $\Delta Q=Q\left(t_{1}\right)-Q\left(t_{2}\right),$ where t_1_ and t_2_ are discharging durations [48]. The change in ΔQ was collected at varying temperatures and electric fields for a constant rate window $\tau_{m}=\left(t_{2}-t_{1}\right)/\ln\left(t_{2}/t_{1}\right)$. The released charge is described as follows [48]:

(3) $$Q\left(t\right)=Q_{0}\;\left[\exp\left(-e_{p}t_{1}\right)-\exp\left(-e_{p}t_{2}\right)\right]$$

where $Q_{0}=\int_{0}^{\infty }Q_{0}\left(t\right)\mathrm{dt}$. For hole trap sites, $e_{p}={\sigma \nu }_{\mathrm{th}}N_{c}\exp\left(-E_{a}/\mathrm{kT}\right)$ is the hole emission rate, $\nu_{\mathrm{th}}=\sqrt{\frac{3\mathrm{kT}}{m_{e}}}$ = the electron thermal velocity, $N_{C}=2{\left(\frac{3\mathrm{kT}}{m_{e}}\right)}^{3/2}$ = the effective density of states in the conduction band, $m_{p}^{*}$ is the effective mass of the hole or electron, and σ is the capture cross section estimated from the intercept of $\ln\left(\tau_{m}^{-1}/{\Gamma T}^{2}\right)$, versus 1000/T [49].

Impedance analysis was performed using an Agilent E4980A (or 4284A) Precision LCR Meter (Santa Rosa, CA, USA). The electric modulus data at 260–340 °C was recorded using a 100 mV AC amplitude at 0.1–10^5^ Hz using a Solartron SI1287 potentiostat with electrochemical interface and 1255B frequency response analyzer.

## 3. Results and Discussion

**Figure 1:**
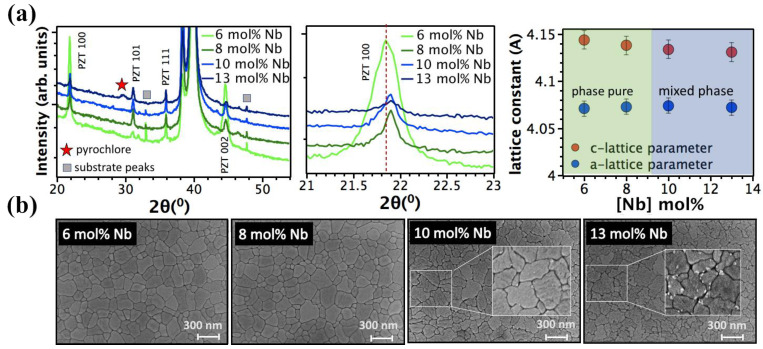
(**a**) XRD diffraction patterns and lattice parameter, (**b**) Top surface FESEM images of 6–13 mol% Nb-doped PZT films prepared using 10 mol% excess PbO.

Figure 1a exhibits the XRD patterns of PNZT films containing different Nb contents.

These films were all prepared with y = 0.1. No second phases were observed in the XRD patterns up to 8 mol% Nb. A XRD peak at 2θ ≈ 29° (100% peak), corresponding to the pyrochlore (or fluorite) phase was observed for Nb levels ≥ 10 mol% (Figure 1a), indicating a Nb solubility limit in PZT of around 8 mol%. This is in good agreement with the FESEM findings (Figure 1b). A pyrochlore phase was observed along the grain boundaries in PZT films with Nb doping levels higher than 8 mol%. This value is comparable with the reported solubility limits of Nb in PZT ceramics [30,31,49]. The kinetics of perovskite formation have been widely reported in the literature; it is nucleation controlled due to rapid growth kinetics. Pyrochlore and/or fluorite phases that are more tolerant to oxygen and lead vacancies and forms if precise stoichiometry is not attained [25,50]. For PZT films, on the other hand, stability of the perovskite phase was found to be strongly influenced by Nb and Pb content, growth temperature, annealing atmosphere, as well as the presence of a PbO seed layer [22,28,34,40,51]. No secondary phase was observed up to 10 mol% Nb in doped PZT films at the MPB (morphotropic phase boundary) composition (Zr/Ti = 52/48) annealed at 650 °C [28]. Increasing the annealing temperature to 700 °C reduced the solubility limit of Nb to levels ≥ 4 mol% due to the formation of a Pb-deficient pyrochlore [34]. With Nb addition, the crystallization temperature needed to attain phase pure perovskite increased [22]. Higher levels of Nb solubility ≥ 13 mol% were observed for sputtered PZT films [41,42,43].

The {100} Lotgering factor, $f_{001}$, for PNZT films was calculated using Equations (4) and (5) to evaluate the texture fraction.

(4) $$f_{001}=\frac{p-p_{0}}{1-p_{0}}$$

where p represents the ratio of the sum of the intensities of {001} peak intensities and the sum of all peak intensities (Equation (2)). $p_{0}$ denotes a similar ratio for a randomly oriented sample. The film with 6 mol% Nb is polycrystalline with a {100} Lotgering factor of 84%, suggesting that the orientation of PNZT films is not governed by (1) the {111} Pt and/or a PbPt_x_ intermetallic phase [52] or

(5) $$p=\frac{\sum I\left\{001\right\}}{\sum I\left(\mathrm{hkl}\right)}$$

(2) the diffusion of Ti through the Pt and subsequent oxidation of Ti to TiO_x_ upon annealing; this oxidation enhances nucleation of (111)-oriented PZT films due to the reduced lattice mismatch between Pt and perovskite [53,54]. It has been reported that Nb doping facilitates {100}-oriented crystal growth of PZT films in the absence of other mechanisms. When homogenous nucleation is dominant, {100}-oriented growth is energetically favored; lower interfacial energy causes rapid growth of {100} nuclei, relative to the other orientations. Increasing Nb concentration up to 4 mol% Nb favors {100}-preferential growth in PZT films [55]. Increasing Nb level to ≥8 mol% suppressed {100}-preferential growth; PZT films present random orientation with a Lotgering factor ($f_{001}$)**,** ranging from 0.25 to 0.54. (Figure 1a). This may be due to (1) increased stability of the transient pyrochlore at higher Nb concentration, which eases the conversion of (222) and (400) pyrochlore to {110} perovskite planes, [25] (2) PbO accumulation along the grain boundaries due to excess lead vacancy formation [56], and/or (3) the presence of pre-existing interfaces that can favor random nucleation.

Figure 1a shows the lattice parameter and c/a ratio of the PNZT films. Changes in the lattice parameters confirmed PNZT solid solution formation. The addition of Nb had multiple effects on the films: out-of-plane lattice parameters decreased slightly, while in-plane lattice parameters remained unchanged, and the c/a ratio and unit cell volume decreased. With the addition of Nb ions, lead vacancies ($V_{Pb}^{\prime \prime }$) were created, removing large lead ions (1.63 Å) and contracting the perovskite lattice. As shown in previous reports [28,31,57], substituting the Nb^5+^ (0.78 Å) ion onto the Zr^4+^ (0.86 Å) site reduced the unit cell volume.

**Figure 2:**
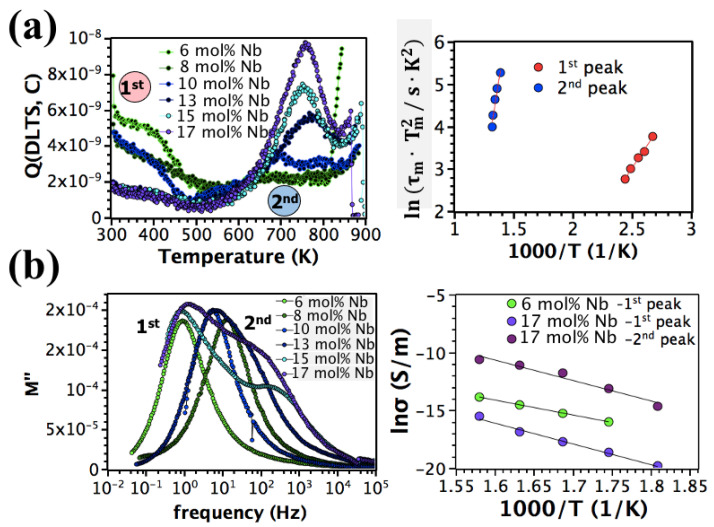
Q-DLTS of (**a**) phase pure 6–8 and mixed phase 10–17 mol% Nb-doped PZT films and the Arrhenius plot of the Q-DLTS signals. (**b**) Imaginary modulus of phase pure 6–8 and mixed phase 10–17 mol% Nb doped PZT films as a function of frequency and a temperature dependence of conductivity.

The effect of Nb doping on the defect chemistry was studied via Q-DLTS and modulus spectroscopy (Figure 2).

Two distinct Q-DLTS peaks are visible between 300 and 900 K (Figure 2a). Activation energies of 0.26 ± 0.04 and 1.4 ± 0.08 eV were recorded for the low and high temperature peaks, respectively. These values were estimated from the slope of the $\ln\left(\tau \cdot T_{m}^{2}\right)$ versus 1000/T. Smyth et al. [20] attributed the 0.26 eV value to hole hopping between Pb^2+^ and Pb^3+^ sites [58]. The activation energy of 1.4 eV was attributed to hole migration between $V_{Pb}^{\prime \prime }$ [59]. The contribution of the pyrochlore phase to the Q-DLTS peaks was neglected, as no Q-DLTS signal was detected in the pyrochlore film (30 mol% Nb-doped PZT) using the same discharging durations and bias levels [45]. The density of these trap levels, which is proportional to the Q-DLTS signal, changes depending on the Nb level in the PZT films; the density of Pb^2+^/Pb^3+^ trap sites decreases, while the $V_{Pb}^{\prime \prime }$ trap level density rises with increasing Nb concentration from 6 to 17 mol%.

The increase in $\left[V_{Pb}^{\prime \prime }\right]$ with Nb concentration was also proved by modulus spectroscopy (Figure 2b). A single modulus peak with an activation energy of 1.1 eV, corresponding to electron trapping by Ti^4+^, was observed for 6 mol% Nb-doped PZT films [60]. With increasing Nb level from 6 to 8 mol%, an activation energy extracted from the slope of the Arrhenius plot increases from 1.1 to 1.4 eV, suggesting that the dominant charge transport mechanism responsible for the bulk film conductivity changes from electron trapping via Ti^4+^ to hole migration between $V_{Pb}^{\prime \prime }$, with increasing Nb concentration. A further increase in the Nb concentration to 13 mol% broadens the modulus peak, and a second peak with a similar activation energy (1.4 eV) emerged at 15 mol% Nb, implying the presence of a heterogeneous conductivity profile, possibly due to a non-uniform distribution of $V_{Pb}^{\prime \prime }$. It was previously demonstrated that Pb-site stoichiometry is non-uniform across the thickness of the 13 mol% Nb-doped PZT films; a noticeable decrease in Pb content was observed in Nb-rich regions of each PZT layer [45].

**Figure 3:**
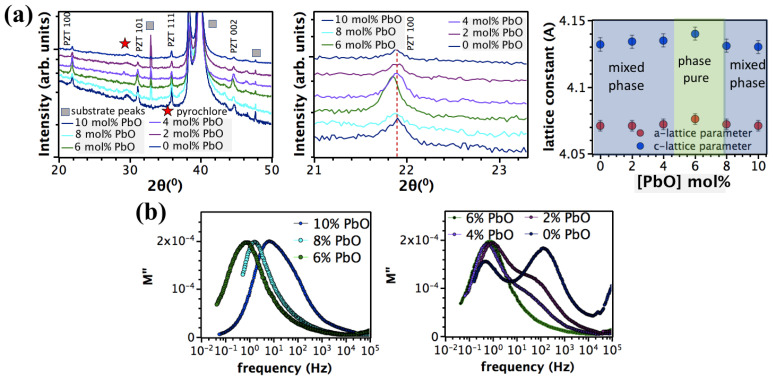
(**a**) XRD diffraction patterns and lattice parameter of 13 mol% Nb-doped PZT films prepared using 0–10 mol% excess PbO. (**b**) Imaginary modulus of 13 mol% Nb-doped PZT films with 0–10 mol% excess PbO level as a function of frequency.

To enhance the solubility limit of Nb in PZT films, assuming that $Nb_{Ti}^{•}$ ions are compensated by $V_{Pb}^{\prime \prime }$, the PbO content in the PZT solution was incrementally decreased from 10 to 0 mol%. Figure 3a shows the XRD patterns of the 13 mol% Nb-doped PZT films containing different PbO excess contents in the precursor.

As expected, the intensity of the XRD peaks, corresponding to the pyrochlore phase, gradually declined on decreasing y from 0.10 to 0.06 and eventually disappeared at y = 0.06; this was accompanied by an increasing intensity in the XRD peaks attributed to the perovskite structure. This clearly indicates that PbO content controls the stability of pyrochlore in 13 mol% Nb-doped PZT films. Additionally, the *a* and *c*-lattice parameters increased as the film became phase pure perovskite at y = 0.06, supporting solid solution formation (Figure 3a). It was previously shown that the solubility limit of Nb ions in PZT 65/35 ceramics increased from 4 to 7 mol% by decreasing the excess PbO content from 4 to 0 mol% [39].

The presence of $V_{Pb}^{\prime \prime }$ in 13 mol% Nb-doped PZT film with 0–10 mol% PbO was also demonstrated by modulus spectroscopy (Figure 3b). For 13 mol% Nb-doped PZT film prepared from solutions with y = 0.1, 0.08, or 0.06, only one modulus peak was observed; it had an activation energy of 1.4 eV, corresponding to hole migration between $V_{Pb}^{\prime \prime }$. On decreasing y from 0.10 to 0.06, (1) the relaxation frequency of the modulus peak shifted to lower frequencies, suggesting that the film conductivity continuously decreased as the film became phase pure perovskite, and (2) the width of the modulus peak diminished, indicating a more uniform distribution of defects, $V_{Pb}^{\prime \prime }$. These findings suggest a plausible charge compensation mechanism for heavily Nb-doped PZT films reported by other researchers [41,42,43,44].

Decreasing y to 0.04 leads to the formation of a pyrochlore phase; the pyrochlore peak becomes more intense as y decreases from 0.04 to 0 (Figure 3a). It is possible that this occurs because the lead vacancy concentration overbalances that needed to compensate the Nb content. The conductivity profile of the film became non-uniform on decreasing y from 0.4 to 0; two modulus peaks with an activation energy of 1.4 eV were observed within the 10^−2^–10^5^ Hz range. It is speculated that this could be due to a non-uniform distribution of lead vacancies across the film. A non-uniform distribution of defects, such as lead vacancies, throughout the film thickness, could arise from segregation of Nb ions into Zr-rich regions in heavily Nb-doped (13 mol%) PZT films with Zr/Ti gradients [61]. A strong lead deficiency was previously reported in Nb- and Zr-rich zones, [45] where $Nb_{Ti}^{•}$ ions are ionically compensated by $V_{Pb}^{\prime \prime }$.

**Figure 4:**
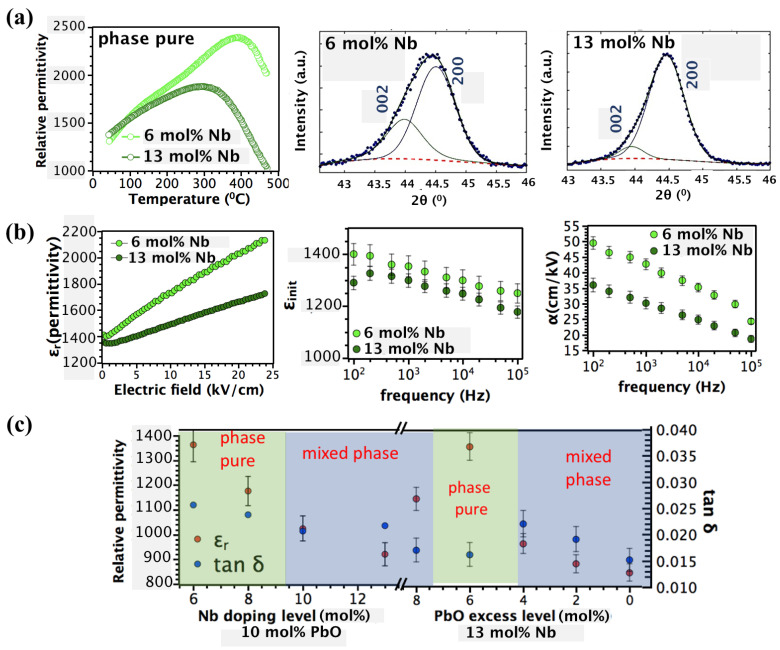
(**a**) Temperature dependence of permittivity and the peak fitting of the 002/200 peaks for the phase pure 6 and 13 mol% Nb-doped PZT films, (**b**) electric field dependence of permittivity, frequency dispersion of Rayleigh parameters, α  and $\varepsilon_{init}$ in phase pure 6 and 13 mol% Nb-doped PZT films, (**c**) variation in relative permittivity, and dielectric loss with Nb and PbO content in phase pure and mixed phase films.

Figure 4a (left panel) shows the temperature dependence of the dielectric constant for phase pure 6 and 13 mol% Nb-doped PZT films.

The Curie temperature, $T_{C}$, diminishes with Nb content, as reported elsewhere [27,30,31]. A lower Curie temperature in 13 mol% Nb-doped PZT influences the stress state and domain configuration of the film. The residual thermal stress builds up upon cooling from the crystallization temperature $T_{\mathrm{crystallization}}$ to $T_{C}$ due to mismatch between thermal expansion coefficient of the substrate and the film that can be described as follows:

(6) $$\sigma_{\mathrm{th}}=\left(\frac{Y_{f}}{1-\nu_{f}}\right)\int_{T_{C}}^{T_{\mathrm{crystallization}}}\left(\propto_{\mathrm{sub}}-\propto_{\mathrm{film}\;}\right)\;\mathrm{dT}$$

where: $\nu_{f}$ and $Y_{f}$ are Poisson’s ratio and Young’s modulus of the film, respectively. A higher level of residual tensile stress in phase pure 13 mol% Nb-doped PZT films favors an in-plane domain state in the film. To explore variations in the domain configuration with Nb level in phase pure PZT films, the {002} XRD peaks were fitted using the pseudo-Voight function in LIPRAS software (Figure 4a, middle and right panel) [62]. The integrated intensity under the 200 and 002 peaks is proportional to the volume fraction of in-plane and out-of-plane domains, respectively. Regarding increasing the Nb level from 6 to 13 mol%, the volume fraction of in-plane domains increases, while that of out-of-plane domain decreases, indicating that higher residual tensile stress in phase pure 13 mol% Nb-doped PZT films preferentially align domains in-plane upon cooling below $T_{C}$.

To understand how variation in the domain state affects the intrinsic and extrinsic electrical properties, Rayleigh measurements were performed from 10^2^–10^5^ Hz (Figure 4b, left panel). $\varepsilon_{\mathrm{init}}$ and α were estimated from the intercept and the slope of ε_r_ vs. E in the Rayleigh regime, respectively (Figure 4b, middle and right panel). $\varepsilon_{\mathrm{init}}$ is higher in 6 mol% Nb-doped PZT films than in 13 mol% Nb-doped PZT films, which might be due to an increase in intrinsic lattice response and/or reversible domain wall motion. A higher volume fraction of *a*-domains in 13 mol% Nb-doped PZT films is expected to increase the contribution of intrinsic lattice response to the permittivity. However, an opposite trend is observed for the permittivity, suggesting that the change in $\varepsilon_{\mathrm{init}}$ is primarily due to reversible domain wall motion.

*α*, representative of irreversible domain wall motion, is also higher in 6 mol% Nb-doped PZT films. The suppression of α in 13 mol% Nb-doped PZT films might be related to (1) reduced 90° domain wall density or/and (2) higher in-plane stresses and/or lead vacancy concentrations that might create deep potential wells in the potential energy landscape and reduce the extrinsic contribution to the permittivity.

Figure 4c illustrates the dielectric constant and loss as a function of Nb and PbO concentration. The $\varepsilon_{r}$ dramatically decreased from 1360 ± 8 to 940 ± 6 on increasing the Nb concentration from 6 to 13 mol%. This is attributed to the appearance of a non-ferroelectric pyrochlore phase detected via FESEM and XRD. When the pyrochlore phase is present as a thin layer on the surface of the film, it creates a lower permittivity capacitor electrically in series with the PZT film. This leads to a strong decrease in the dielectric constant at higher Nb doping levels. A similar deterioration in dielectric properties with Nb content was previously reported [22,31,63,64]. No significant change is observed in dielectric loss with Nb content; the dielectric loss, tanδ, varies from 0.18 to 0.25. The degradation in dielectric properties of heavily Nb-doped (13 mol%) mixed phase PZT films was regained by optimizing the excess PbO level. When y decreased from 0.10 to 0.06, ε_r_ increased to 1330 ± 9. However, a further decrease in solution PbO content induced reduction in ε_r_ due to the presence of pyrochlore phase.

**Figure 5:**
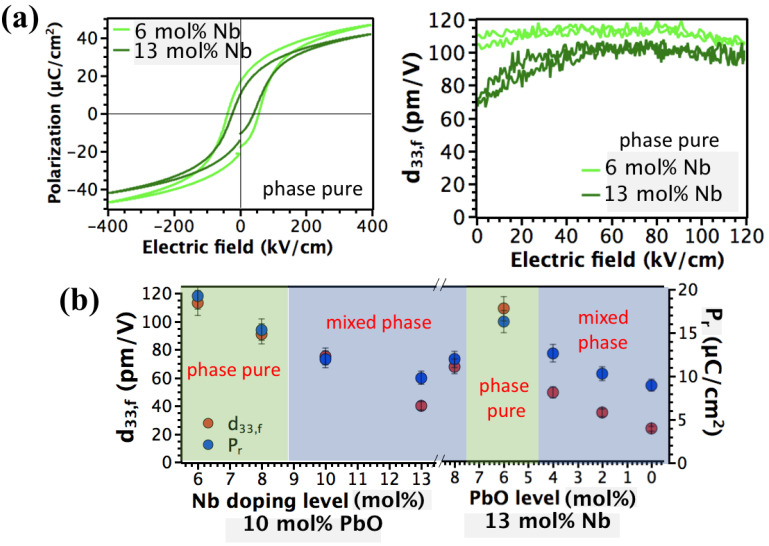
(**a**) Change in longitudinal piezoelectric coefficient d_33,f_ and remanent polarization, P_r_, with Nb and PbO level in PZT films, (**b**) P-E hysteresis loops and the longitudinal piezoelectric coefficient d_33,f_ of phase pure 6 and 13 mol% Nb-doped PZT films.

The variation in the d_33,f_ and P_r_ with Nb and PbO level in PZT films are shown in Figure 5a.

For films containing 6 mol% Nb, the remanent polarization is 26 µC/cm^2^; it decreased to 22, 20, and 16 µC/cm^2^ for 8, 10, and 13 mol% Nb levels, respectively. The large increase in the volume fraction of pyrochlore with higher dopant levels reduces the remanent polarization. Similarly, the maximum value of the d_33,f_ observed was smaller for higher Nb doping levels. For 6% Nb-doped PZT film, the remanent d_33,f_ value after poling at 180 °C was 112 ± 4 pm/V, decreasing to 86 ± 3, 64 ± 3, and 42 ± 3 pm/V for Nb doping levels of 8, 10, and 13 mol%, respectively. The severe degradation in piezoelectric properties is a consequence of the increased quantity of the non-polar pyrochlore phase. These findings are in agreement with XRD and FESEM results.

The deterioration in ferroelectric properties was partially recovered by decreasing the excess PbO content in the solution (Figure 5a). The remanent polarization of the 13 mol% Nb-doped PZT film increased from 16 to 22 µC/cm^2^ on decreasing y from 0.10 to 0.06. Likewise, the remanent piezoelectric constant of 13% Nb-doped PZT increased more than two times on decreasing y from 0.10 to 0.06; the remanent d_33,f_ were 42 ± 3 and 106 ± 4 pm/V, respectively. The improvement in piezoelectric properties is mainly due to increased stability of the perovskite phase due to the enhanced solubility of Nb. For y *<* 0.06, both the remanent polarization and the remanent d_33,f_ decreased significantly due to reoccurrence of the pyrochlore phase.

Figure 5b shows the P-E hysteresis and the d_33,f_ of phase pure 13 mol% Nb-doped PZT films. Prior to the d_33,f_ measurement, the sample was poled under an electric field of 250 kV/cm at 180 °C for 30 min, and the d_33,f_ was recorded under a unipolar electric field parallel to the poling direction in 0–120 kV/cm. When increasing Nb level from 6 to 13 mol% in the PZT film, the P_r_ and d_33,f_ decreased from 26 to 22 µC/cm^2^ and 112 to 106 pm/V, respectively. This might be attributed to (1) a higher volume fraction of out-of-plane polarization and (2) increased ferroelastic domain wall density. The coercive field also depends on the doping level in phase pure films; it drastically decreases at higher Nb doping levels, consistent with a softening of the lattice [45].

**Figure 6:**
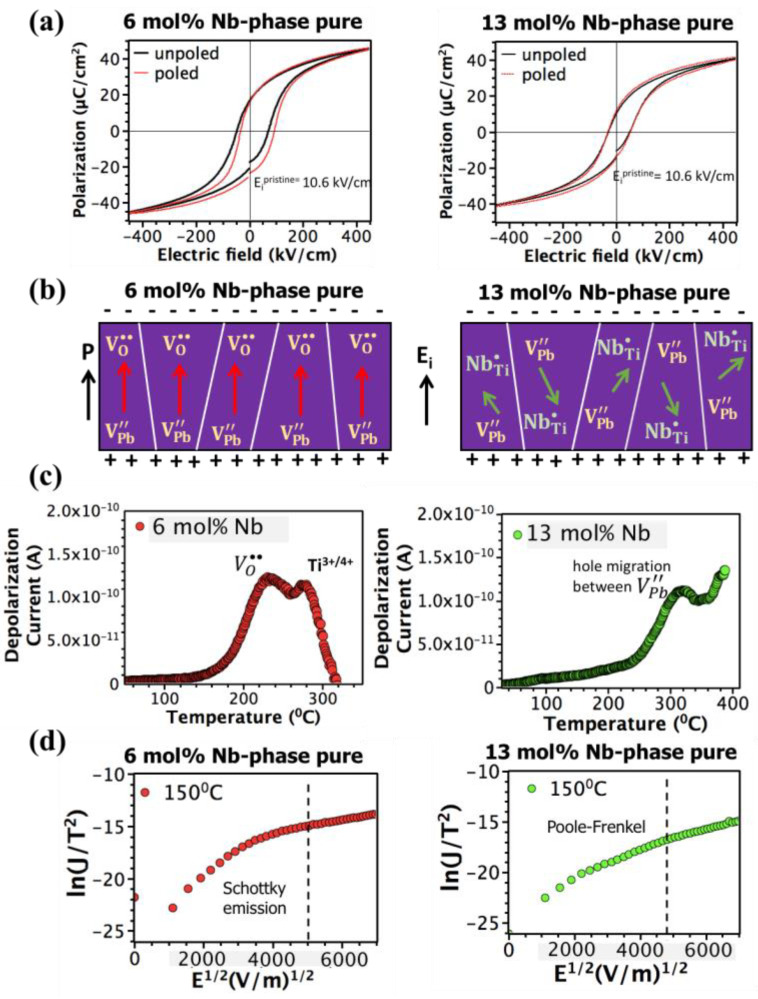
(**a**) P-E Hysteresis loops of phase pure 6 and 13 mol% Nb-doped PZT films before and after poling at 150 °C under an electric field of 200 kV/cm for 40 min. (**b**) Schematic picture of imprint mechanisms observed in 6 and 13 mol% Nb-doped PZT films. (**c**) Temperature dependence of depolarization current in 6 and 13 mol% Nb-doped PZT films exposed to an electric field of 300 kV/cm at 180 °C for 3 h. (**d**) Electric field dependence of leakage current in phase pure 6 and 13 mol% Nb-doped PZT films.

The impact of 13 mol% Nb on self-imprint in the films is demonstrated in Figure 6a. The imprint can be described as formation of a preferred orientation state due to a shift along the field axis in the hysteresis loop (Equation (7)). Previous studies reported

(7) $$E_{i}=\frac{+E_{c}-\left|-E_{c}\right|}{2}$$

imprint in sputtered Nb-doped PZT films (≥10 mol%) and attributed this to (1) the alignment of ${\left(V_{Pb}^{\prime \prime }-Nb_{Ti}^{•}\;\right)}^{\prime }$ defect dipoles aligned by a residual stress gradient and (2) the presence of oppositely charged $V_{Ti,Zr}^{\prime \prime \prime \prime }$ and $Nb_{Ti}^{•}$ defects [42,44]. Here, however, a much smaller internal field (calculated using Equation (4)) was observed for the 13 mol% Nb-doped PZT films prepared by chemical solution deposition. No significant difference in the degree of imprint was observed as a function of the [Nb] in the pristine state; the amplitude of the internal field was found to be 10 and 10.5 kV/cm for phase pure 6 and 13 mol% Nb, respectively. This suggests that high Nb doping itself is not responsible for the high degree of self-imprint observed in sputtered PNZT films (12–13 mol% Nb) [42,44].

To further investigate the formation of imprint, the films were poled under an applied electric field of 200 kV/cm at 150 °C for 40 min. Intriguingly, a much higher level of imprint (30 kV/cm) was introduced in phase pure 6 mol% Nb-doped PZT films than 13 mol% Nb-doped PZT films (11.5 kV/cm). This suggests that the mechanism responsible for the formation of a built-in internal field differs in 6 and 13 mol% Nb-doped PZT films. Different physical mechanisms, including defect dipole alignment, stress gradient due to an asymmetric electrode structure, a space charge, and charge trapping, were proposed to account for imprint in the literature [19,65,66].

The schematic that describes the difference in imprint mechanism in phase pure 6 and 13 mol% Nb-doped PZT films is shown in Figure 6b. In 6 mol% Nb-doped PZT films, the only possible re-orientable defect dipole is ${\left(V_{Pb}^{\prime \prime }-V_{O}^{••}\;\right)}^{x}$ due to PbO evaporation upon annealing. Oxygen vacancies are highly mobile in perovskite compounds. Thus, alignment of these defect dipoles, as a result of short-range oxygen vacancy migration, can contribute to the built-in internal field. To prove the presence of mobile $V_{O}^{••}$ in 6 mol% Nb-doped PZT films, TSDC measurements were conducted after poling the films under 300 kV/cm at 180 °C for 12 h. No TSDC peak, corresponding to a ${\left(V_{Pb}^{\prime \prime }-V_{O}^{••}\;\right)}^{x}$ defect dipole, was detected, presumably due to dissociation of these defect dipoles under long poling times, which was needed to make the signal detectable over the background pyroelectric current (Figure 6c, left panel). A low temperature TSDC peak with an activation energy of 0.6 eV confirms the presence of $V_{O}^{••}$. The high temperature TSDC peak, on the other hand, has an activation energy of 1.1 eV that corresponds to electron trapping via Ti^4+^. In addition to the alignment of ${\left(V_{Pb}^{\prime \prime }-V_{O}^{••}\;\right)}^{x}$, electronic charges can contribute to the internal field [20]. Schottky emission is the dominant conduction mechanism in 6 mol% Nb-doped PZT films at the electric fields (200 kV/cm) and temperatures (150 °C) used to introduce imprint (Figure 6d, left panel). It was previously shown that electrons are the majority carriers in Nb-doped PZT films [21]. Both TSDC and modulus spectroscopy results confirm the presence of Ti^3+/4+^. Thus, it is believed that for 6 mol% Nb-doped CSD (Chemical Solution Deposition) derived PZT films, the internal field formation is primarily governed via (1) the alignment of ${\left(V_{Pb}^{\prime \prime }-V_{O}^{••}\;\right)}^{x}$ and (2) the injection and subsequent electron trapping by Ti^4+^.

In contrast to 6 mol% Nb-doped PZT films, no TSDC peak attributed to electromigration of $V_{O}^{••}$ was observed in phase pure 13 mol% Nb-doped PZT films. Instead, a high temperature peak with an activation energy of 1.4 eV was found; it is associated with hole migration between $V_{Pb}^{\prime \prime }$, in good agreement with the electric modulus spectroscopy results (Figure 6c, right panel). $V_{Pb}^{\prime \prime }$ is immobile at room temperature in PZT films, as it requires an activation energy higher than 2 eV [18]. The lack of mobile $V_{O}^{••}\;,$ together with the presence of immobile $V_{Pb}^{\prime \prime }$ in 13 mol% Nb-doped PZT films, is likely to be responsible for the lower internal field formation upon thermal poling. Furthermore, immobile defect complexes, such as ${\left(Nb_{Ti}^{•}-V_{Pb}^{\prime \prime }-Nb_{Ti}^{•}\;\right)}^{x}$, can be created in heavily Nb-doped PZT films, which suppresses the contribution of defect dipole alignment to imprint in these films. 

To assess the contribution of electronic conduction to internal field formation, I–V results were fitted to explore the dominant conduction mechanism at electric field and temperature range used to introduce imprint. The permittivity of 13 mol% Nb-doped PZT films at optical frequencies was estimated from the slope of a Poole-Frenkel plot (Figure 6d, right panel). The estimated refractive index of 2.4 is in agreement with the refractive index of PZT (2.25). Hence, the dominant conduction mechanism is Poole-Frenkel emission, mainly due to hole migration between $V_{Pb}^{\prime \prime }$, as demonstrated by TSDC and Q-DLTS results. Since the dominant conduction mechanism at the electric field and temperature range used to introduce imprint is Poole-Frenkel emission, hole migration between $V_{Pb}^{\prime \prime }$ could plausibly contribute to internal field formation.

## 4. Conclusions

Nb-doped Pb_1+y_(Zr_0.52_Ti_0.48_)_1−x_Nb_x_O_3_, x = 0.06–0.13, y = 0–0.1 thin films on Pt metallized silicon substrates were produced by chemical solution deposition. Nb doping and PbO excess level were found to have a strong influence on the phase development and electrical responses of PZT films. Pyrochlore-free PZT films with phase-pure perovskite structure were obtained up to 8 mol% Nb from solutions with y = 0.1. Nb concentrations exceeding 8 mol% resulted in pyrochlore formation in the PZT thin films. The quantity of pyrochlore increased with higher Nb concentrations and led to rapid degradation of dielectric and ferroelectric properties. The solubility limit of Nb in PZT films is improved by decreasing y from 0.1 to 0.06 in 13 mol% Nb-doped PZT films. That is, ionic charge compensation of $Nb_{Ti}^{•}$ ions was accomplished by increased lead vacancy concentrations ($Nb_{Ti}^{•}$ ≈ 2 $V_{Pb}^{\prime \prime }$) in the perovskite lattice; this enhanced the perovskite structure stability. XRD results confirmed that no second phase developed in 13 mol% Nb-doped PZT films with y *=* 0.06. This leads to an increase in the dielectric and piezoelectric properties of 13 mol% Nb-doped PZT films; the remanent d_33,f_ increased more than two times to 106 ± 4 pm/V for y = 0.06. Similarly, ε_r_ increased from 941 ± 6 to 1330 ± 9 at the same composition. It was shown that the amplitude of the internal field was much higher in phase pure 6 mol% Nb-doped PZT films (30 kV/cm) compared to phase pure 13 mol% Nb-doped PZT films (11.5 kV/cm) after poling at 150 °C under an electric field of 200 kV/cm for 40 min. The enhanced internal field at lower Nb levels was attributed to the presence of highly mobile oxygen vacancies. Two different mechanisms might contribute to the development of internal field in 6 mol% Nb-doped PZT: (1) alignment of ${\left(V_{Pb}^{\prime \prime }-V_{O}^{••}\;\right)}^{x}$ defect dipoles and (2) electron injection and subsequent trapping by Ti^4+^. At 13 mol% Nb, the [$V_{O}^{••}]$ significantly diminished, while immobile $\left[V_{Pb}^{\prime \prime }\right]$ rose, which led to the lower level of internal field in heavily Nb-doped PZT films. Additionally, immobile defect complexes, such as ${\left(Nb_{Ti}^{•}-V_{Pb}^{\prime \prime }-Nb_{Ti}^{•}\;\right)}^{x}$, cannot contribute substantively to the development of imprint in these films. The main mechanism that governs development of internal field in 13 mol% Nb-doped PZT was found to be hole migration between $V_{Pb}^{\prime \prime }$.

## Data Availability

The data that support the findings of this study are available upon request from the corresponding author.

## Supplementary Materials

The following supporting information can be downloaded at: https://www.mdpi.com/article/10.3390/ma16113970/s1, Figure S1: Variation in pyrochlore structure with 48f oxygen parameter in A_2_B_2_O_6_O′.

## References

[1] Muralt P., Polcawich R.G., Trolier-McKinstry S. (2009). Piezoelectric thin films for sensors, actuators, and energy harvesting. MRS Bull..

[2] Muralt P., Ledermann N., Baborowski J., Barzegar A., Gentil S., Belgacem B., Petitgrand S., Bosseboeuf A., Setter N. (2005). Piezoelectric micromachined ultrasonic transducers based on PZT thin films. IEEE Trans. Ultrason. Ferroelectr. Freq. Control.

[3] Funakubo H., Dekkers M., Sambri A., Gariglio S., Shklyarevskiy I., Rijnders G. (2012). Epitaxial PZT films for MEMS printing applications. MRS Bull..

[4] Yeo H.G., Ma X., Rahn C., Trolier-McKinstry S. (2016). Efficient piezoelectric energy harvesters utilizing (001) textured bimorph PZT films on flexible metal foils. Adv. Funct. Mater..

[5] Jaffe B., Cook W.R., Jaffe H. (1971). Piezoelectric Ceramics.

[6] Tuttle B.A., Doughty D.H., Schwartz R.W., Garino T.J., Martinez S.L., Goodnow D., Tissot R.G., Hammetter W.F. (1999). Chemically prepared PZT films with niobium additions. Ceram. Trans..

[7] Nguyen M.D., Houwman E.P., Dekkers M., Rijnders G. (2017). Strongly enhanced piezoelectric response in lead zirconate titanate films with vertically aligned columnar grains. ACS Appl. Mater. Interfaces.

[8] Otani Y., Fukuda Y., Okamura S., Nakamura K., Nishida T., Uchiyama K., Shiosaki T. (2009). Formation mechanism of oriented Pb(Zr, Ti)O_3_ thin films on platinum bottom electrodes from amorphous films prepared by liquid delivery metalorganic chemical vapor deposition. Jpn. J. Appl. Phys..

[9] Bai W., Meng X.J., Lin T., Tian L., Bing C., Liu W.J., Ma J.H., Sun J.L., Chu J.H. (2009). Effect of Fe-doping concentration on microstructure, electrical, and magnetic properties of Pb(Zr_0.5_Ti_0.5_)O_3_ thin films prepared by chemical solution deposition. J. Appl. Phys..

[10] Zhang Q., Whatmore R.W. (2003). Improved ferroelectric and pyroelectric properties in Mn-doped lead zirconate titanate thin films. J. Appl. Phys..

[11] Koh D., Ko S.W., Yang J.I., Akkopru-Akgun B., Trolier-McKinstry S. (2022). Effect of Mg-doping and Fe-doping in lead zirconate titanate (PZT) thin films on electrical reliability. J. Appl. Phys..

[12] Kozielski L., Adamczyk M., Erhart J., Pawełczyk M. (2012). Application testing of Sr doping effect of PZT ceramics on the piezoelectric transformer gain and efficiency proposed for MEMS actuators driving. J. Electroceramics.

[13] Zhang M.F., Wang Y., Wang K.F., Zhu J.S., Liu J.-M. (2009). Characterization of oxygen vacancies and their migration in Ba-doped Pb (Zr_0.52_Ti_0.48_)O_3_ ferroelectrics. J. Appl. Phys..

[14] Sheng T., Narayanan M., Beihai M., Shanshan L., Koritala R.E., Balachandran U., Donglu S. (2013). Effect of lanthanum content and substrate strain on structural and electrical properties of lead lanthanum zirconate titanate thin films. Mater. Chem. Phys..

[15] Atkin R.B., Holman R.L., Fulrath R.M. (1971). Substitution of Bi and Nb ions in lead zirconate–titanate. J. Am. Ceram. Soc..

[16] Choi W.-Y., Ahn J.-H., Lee W.J., Kim H.-G. (1998). Electrical properties of Sb- doped PZT films deposited by d.c. reactive sputtering using multi-targets. Mater. Lett..

[17] Robels U., Arlt G. (1993). Domain wall clamping in ferroelectrics by orientation of defects. J. Appl. Phys..

[18] Morozov M.I., Damjanovic D. (2010). Charge migration in Pb(Zr,Ti)O_3_ ceramics and its relation to ageing, hardening, and softening. J. Appl. Phys..

[19] Grossmann M., Lohse O., Bolten D., Boettger U., Schneller T. (2002). The interface screening model as origin of imprint in PbZr_x_Ti_1-x_O_3_ thin films. I. dopant, illumination, and bias dependence. J. Appl. Phys..

[20] Akkopru-Akgun B., Zhu W., Lanagan M.T., Trolier-McKinstry S. (2019). The effect of imprint on remanent piezoelectric properties and ferroelectric aging of Mn or Nb doped PbZr_0.52_Ti_0.48_O_3_ thin films. J. Am. Ceram. Soc..

[21] Akkopru-Akgun B., Bayer T., Tsuji K., Wang K., Randall C.A., Lanagan M.T., Trolier-McKinstry S. (2021). Leakage current characteristics and DC resistance degradation mechanisms in Nb doped PZT films. J. Appl. Phys..

[22] Haccart T., Remiens D., Cattan E. (2003). Substitution of Nb doping on the structural, microstructural and electrical properties in PZT films. Thin Solid Films.

[23] Kim W.S., Ha S.-M., Park H.-H., Kim C.E. (1999). The effects of cation-substitution on the ferroelectric properties of sol-gel derived PZT thin film for FRAM application. Thin Solid Films.

[24] Zhu W., Fujii I., Ren W., Trolier-McKinstry S. (2012). Domain wall motion in A and B site donor-doped Pb(Zr_0.52_Ti_0.48_)O_3_ Films. J. Am. Ceram. Soc..

[25] Kwok C.K., Desu S.B. (1992). Pyrochlore to perovskite phase transformation in sol-gel derived lead- zirconate-titanate thin films. Appl. Phys. Lett..

[26] Souza E.C.F., Simões A.Z., Cilense M., Longo E., Varela J.A. (2004). The effect of Nb doping on ferroelectric properties of PZT thin films prepared from polymeric precursors. Mater. Chem. Phys..

[27] Paiva-Santos C.O., Oliveira C.F., Las W.C., Zaghete M.A., Varela J.A., Cilense M. (2000). Effect of niobia on the crystal structure and dielectric characteristics of Pb(Zr_0.45_Ti_0.55_)O_3_ prepared from polymeric precursor. Mater. Res. Bull..

[28] Kurchnia R., Milne S.J. (2003). Effect of niobium modifications to PZT (53/47) thin films made by a sol-gel route. J. Sol-Gel Sci. Technol..

[29] Araujo E.B., Eiras J.A. (2002). Structural, dielectric and ferroelectric properties of Nb-doped PZT thin films produced by oxide precursor method. Ferroelectrics.

[30] Pereira M., Peixoto A.G., Gomes M.J.M. (2001). Effect of Nb doping on the microstructural and electrical properties of the PZT ceramics. J. Eur. Ceram. Soc..

[31] Mohiddon M.A., Kumar R., Goel P., Yadav K.L. (2007). Effect of Nb doping on structural and electric properties of PZT (65/35) ceramic. IEEE Trans. Dielectr. Electr. Insul..

[32] Chu S.-Y., Chen T.-Y., Tsai I.-T., Water W. (2004). Doping effects of Nb additives on the piezoelectric and dielectric properties of PZT ceramics and its application on SAW device. Sens. Actuators A Phys..

[33] Li Q., Wang X., Wang F., Dou J., Xu W., Zou H. (2017). Effect of Nb doping on crystalline orientation, electric and fatigue properties of PZT thin films prepared by sol-gel process. J. Ceram. Sci. Technol..

[34] Klissurska R.D., Brooks K.G., Reaney I.M., Pawlaczyk C., Kosec M., Setter N. (1995). Effect of Nb doping on the microstructure of sol-gel derived PZT thin films. J. Am. Ceram. Soc..

[35] Fan H., Park G.-T., Choi J.-J., Kim H.-E. (2002). Preparation and characterization of sol–gel-derived lead magnesium niobium titanate thin films with pure perovskite phase and lead oxide cover coat. J. Am. Ceram. Soc..

[36] Lee C., Kawano S., Itoh T., Suga T. (1996). Characteristics of sol-gel derived PZT thin films with lead oxide cover layers and lead titanate interlayers. J. Mater. Sci..

[37] Subramanian M.A., Ardvamudan G., Subba Rao G.V. (1983). Oxide oyrochlores—A review. Prog. Solid State Chem..

[38] Klissurska R.D., Tagantsev A.K., Brooks K.G., Setter N. (1997). Use of ferroelectric hysteresis parameters for evaluation of niobium effects in lead zirconate titanate thin films. J. Am. Ceram. Soc..

[39] M’peko J.-C., Peixoto A.G., Jiménez E., Gaggero-Sager L.M. (2005). Electrical properties of Nb-doped PZT 65/35 ceramics: Influence of Nb and excess PbO. J. Electroceramics.

[40] Es-Souni M., Piorra A., Solterbeck C.-H., Iakovlev S., Abed M. (2002). Microstructure and properties of solution deposited Nb-doped PZT thin films. J. Electroceramics.

[41] Fujii T., Hishinuma Y., Mita T., Arakawa T. (2009). Preparation of Nb doped PZT film by RF sputtering. Solid State Commun..

[42] Berenov A., Petrov P., Moffat B., Phair J., Allers L., Whatmore R.W. (2021). Pyroelectric and photovoltaic properties of Nb-doped PZT thin films. APL Mater..

[43] Yang J.-S., Kang Y., Kang I., Lim S., Shin S.-J., Lee J., Hur K.H. (2017). Comparison of the thermal degradation of heavily Nb-doped and normal PZT thin films. IEEE Trans. Ultrason. Ferroelectr. Freq. Control.

[44] Han C.S., Park K.S., Choi H.J., Cho Y.S. (2017). Origin of in situ domain formation of heavily Nb-doped Pb(Zr,Ti)O_3_ thin films sputtered on Ir/TiW/SiO_2_/Si substrates for mobile sensor applications. ACS Appl. Mater. Interfaces.

[45] Akkopru-Akgun B., Wang K., Trolier-McKinstry S. (2022). Links between defect chemistry, conduction, and lifetime in heavily Nb doped lead zirconate titanate films. Appl. Phys. Lett..

[46] Ozer N., Sands T. (2000). Preparation and optical characterization of sol-gel deposited Pb(Zr_0.45_Ti_0.55_)O_3_ Films. J. Sol-Gel Sci. Technol..

[47] Benkler M., Paul F., Schott J., Hanemann T. (2014). Ferroelectric thin film fabrication by direct UV-lithography. Microsyst. Technol..

[48] Lang D.V. (1974). Deep-level transient spectroscopy: A new method to characterize traps in Semiconductors. J. Appl. Phys..

[49] Pintilie L., Pereira M., Gomes M.J.M., Boerasu I. (2004). Pyroelectric current spectroscopy: Example of application on Nb doped Pb (Zr_0.92_Ti_0.08_)O_3_ ceramic for infrared detection. Sens. Actuators A Phys..

[50] Muralt P. (1997). Piezoelectric thin films for MEMS. Integr. Ferroelectr..

[51] Fox G.R., Krupanidhi S.B. (1994). Dependence of perovskite/pyrochlore phase formation on oxygen stoichiometry in PLT thin films. J. Mater. Res..

[52] Chen S., Chen I. (1994). Phase transformations of oriented Pb (Zr_1−x_Ti_x_)O_3_ thin films from Metallo-Organic Precursors. Ferroelectrics.

[53] Bouregba R., Poullain G., Vilquin B., Murray H. (2000). Orientation control of textured PZT thin films sputtered on silicon substrate with TiO_x_ seeding. Mater. Res. Bull..

[54] Brooks K.G., Reaney I.M., Klissurska R., Huang Y., Bursill L., Setter N. (1994). Orientation of rapid thermally annealed lead zirconate titanate thin films on (111) Pt substrates. J. Mater. Res..

[55] Sreenivas K., Reaney I., Maeder T., Setter N., Jagadish C., Elliman R.G. (1994). Investigation of Pt/Ti bilayer metallization on silicon for ferroelectric thin film integration. J. Appl. Phys..

[56] Ryder D.F., Raman N.K. (1992). Sol-gel processing of Nb-doped Pb(Zr, Ti)O_3_ thin films for ferroelectric memory applications. J. Electron. Mater..

[57] Shakeri A., Abdizadeh H., Golobostanfard M.R. (2016). Fabrication of Nb-doped lead zirconate titanate thick films synthesized by sol–gel dip coating method. J. Mater. Sci. Mater. Electron..

[58] Robertson J., Warren W.L., Tuttle B.A., Dimos D., Smyth D.M. (1993). Shallow Pb^3+^ hole traps in lead zirconate titanate ferroelectrics. Appl. Phys. Lett..

[59] Dih J.J., Fulrath R.M. (1978). Electrical conductivity in lead zirconate-titanate ceramics. J. Am. Ceram. Soc..

[60] Raymond M.V., Smyth D.M. (1996). Defects and charge transport in perovskite ferroelectrics. J. Phys. Chem. Solids.

[61] Akkopru-Akgun B., Zhu W., Randall C.A., Lanagan M.T., Trolier-McKinstry S. (2019). Polarity dependent DC resistance degradation and electrical breakdown in Nb doped PZT films. APL Mater..

[62] Esteves G., Ramos K., Fancher C.M., Jones J.L. (2017). LIPRAS: Line-Profile Analysis Software. https://www.researchgate.net/publication/316985889_LIPRAS_Line-Profile_Analysis_Software?channel=doi&linkId=592f83000f7e9beee7619156&showFulltext=true.

[63] Sun H., Zhang Y., Liu X., Guo S., Liu Y., Chen W. (2015). The effect of Mn/Nb doping on dielectric and ferroelectric properties of PZT thin films prepared by sol–gel process. J. Sol-Gel Sci. Technol..

[64] Kayasu V., Ozenbas M. (2009). The effect of Nb doping on dielectric and ferroelectric properties of PZT thin films prepared by solution deposition. J. Eur. Ceram. Soc..

[65] Erdem E., Eichel R.-A., Kungl H., Hoffmann M.J., Ozarowski A., Van Tol J., Brunel L.C. (2008). Characterization of $({Fe}_{Zr,Ti}^{\prime }-V_{O}^{**})*$ defect dipoles in (La,Fe)-codoped PZT 52.5/47.5 piezoelectric ceramics by multifrequency electron paramagnetic resonance spectroscopy. IEEE Trans. Ultrason. Ferroelectr. Freq. Control.

[66] Kim S.H., Kim D.J., Hong J.G., Streiffer S.K., Kingon A.I. (1999). Imprint and fatigue properties of chemical solution derived Pb_1−x_La_x_(ZryTi_1−y_)_1−x/4_O_3_ thin films. J. Mater. Res..

