# Supplementary material for: Tuning Nb Solubility, Electrical Properties, and Imprint through PbO Stoichiometry in PZT Films
Source: Materials (Basel). 2023 May 25;16(11):3970. doi: 10.3390/ma16113970 (PMC10254031; doi:10.3390/ma16113970)
Supplement: Supplementary file 1 [file materials-16-03970-s001.zip › materials-2391079-supplementary.pdf]

Supplementary Material

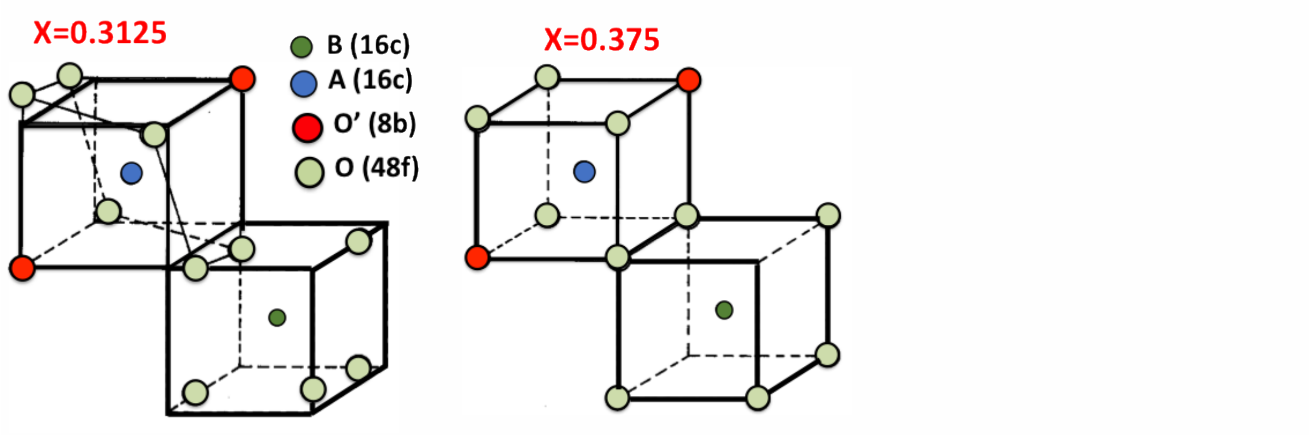

Figure S1. Variation in pyrochlore structure with 48f oxygen parameter in $A_2B_2O_6O'$. This figure was reproduced from [37].
